# Laboratory Validation of Inertial Body Sensors to Detect Cigarette Smoking Arm Movements

**DOI:** 10.3390/electronics3010087

**Published:** 2014-02-27

**Authors:** Bethany R. Raiff, Çağdaş Karataş, Erin A. McClure, Dario Pompili, Theodore A. Walls

**Affiliations:** 1 Department of Psychology, Rowan University, Glassboro, NJ 08028, USA; 2 Department of Electrical and Computer Engineering, Rutgers University, New Brunswick, NJ 08901, USA; 3 Clinical Neuroscience Division, Medical University of South Carolina, Charleston, SC 29425, USA; 4 Department of Psychology, University of Rhode Island, Kingston, RI 02881, USA

**Keywords:** inertial sensors, smoking, edge-detection method, SVM-method

## Abstract

Cigarette smoking remains the leading cause of preventable death in the United States. Traditional in-clinic cessation interventions may fail to intervene and interrupt the rapid progression to relapse that typically occurs following a quit attempt. The ability to detect actual smoking behavior in real-time is a measurement challenge for health behavior research and intervention. The successful detection of real-time smoking through mobile health (mHealth) methodology has substantial implications for developing highly efficacious treatment interventions. The current study was aimed at further developing and testing the ability of inertial sensors to detect cigarette smoking arm movements among smokers. The current study involved four smokers who smoked six cigarettes each in a laboratory-based assessment. Participants were outfitted with four inertial body movement sensors on the arms, which were used to detect smoking events at two levels: the puff level and the cigarette level. Two different algorithms (Support Vector Machines (SVM) and Edge-Detection based learning) were trained to detect the features of arm movement sequences transmitted by the sensors that corresponded with each level. The results showed that performance of the SVM algorithm at the cigarette level exceeded detection at the individual puff level, with low rates of false positive puff detection. The current study is the second in a line of programmatic research demonstrating the proof-of-concept for sensor-based tracking of smoking, based on movements of the arm and wrist. This study demonstrates efficacy in a real-world clinical inpatient setting and is the first to provide a detection rate against direct observation, enabling calculation of true and false positive rates. The study results indicate that the approach performs very well with some participants, whereas some challenges remain with participants who generate more frequent non-smoking movements near the face. Future work may allow for tracking smoking in real-world environments, which would facilitate developing more effective, just-in-time smoking cessation interventions.

## 1. Introduction

Cigarette smoking remains the number one cause of preventable morbidity and mortality in the United States [1]. Each year, one third to one half of smokers attempt to quit at least once [2]; however, approximately 70% of quit attempts fail [3]. Research suggests that an overwhelming majority of smokers (95%) who “slip” (*i.e*., lapse, which could involve smoking only a few puffs from a cigarette) during a quit attempt eventually relapse (*i.e*., return to pre-quit smoking levels; [4,5]). There is a clear need for more research to understand why individuals continue to smoke, why they have difficulty quitting, what leads to a lapse or relapse, and what helps smokers maintain abstinence.

A number of methods are used to estimate smoking status in research and treatment methods, such as: (1) self-reports of smoking status at specific time points [6]; (2) self-reports of smoking in the natural environment at the time that each cigarette is smoked (e.g., ecological momentary assessment; [7]; (3) expired breath carbon monoxide (CO; [8,9]); and (4) metabolites of nicotine found in the urine, saliva, and blood plasma of smokers, such as cotinine [8,10,11]. All of these techniques suffer from limitations in accurately detecting smoking status and patterns of use, which may contribute to the deficits in existing smoking cessation interventions. Although self-reports of smoking are an acceptable method for determining smoking status in many contexts [6], they can suffer from issues such as recall bias [7] and falsification [12] (e.g., incentive-based interventions, pregnant women). Ecological momentary assessment (EMA) is intended to collect information about individual smoking events as they occur in the natural environment; however, EMA suffers from the same potential limitations that exist for other self-report measures described above and requires high levels of participant compliance. Objective, biochemical measures indicating smoking also suffer from limitations. CO is complicated by the fact that it has a short half-life and must therefore be collected frequently to capture smoking events [8]. CO can also be elevated by other sources (e.g., marijuana, car exhaust, second-hand smoke). Tobacco metabolites, such as cotinine, have much longer half-lives than CO, but this can also be problematic because it can be difficult to distinguish recent from past smoking. Also, some metabolites are unable to differentiate nicotine obtained from non-smoking sources (e.g., nicotine replacement therapy, smokeless tobacco, *etc*.). Furthermore, biochemical measures of abstinence do not capture individual smoking events, instead they provide a summary of smoking, and occasionally fail to identify very low levels of smoking [8].

No single measure of smoking status in the natural environment is likely to be 100% accurate. Nevertheless, new efforts to track smoking in real-time are important on their own and in some cases may be combined with existing methods to improve detection and overcome barriers. One solution to the limitations of current methods of assessing smoking status, and in capturing a lapse or relapse when it occurs, is to use a remote monitoring system capable of detecting cigarette smoking, even if only a few puffs of a cigarette are taken in the natural environment. Remotely monitoring health behavior and outcomes is an emerging area within the mobile health (mHealth) field [13]. MHealth is a developing area of innovation, research, and dissemination that incorporates mobile technology, such as mobile devices, health-related applications (apps) for mobile platforms, remote monitoring, body sensors, *etc*. [14]. The goal of this work is to improve health-related research, treatment delivery and fidelity, frequency of data collection, dissemination, just-in-time interventions, respondent burden and cost-effectiveness. This area is particularly exciting for smokers, as many treatment interventions are not successful and smokers progress from lapse to relapse very quickly, all of which could be improved with mobile technology.

There are systems that have been used to detect individual puffs. For example, the Clinical Research Support Systems (CReSS) Pocket device (Borgwaldt KC, Inc., Richmond, VA, USA) has been used in the laboratory to capture individual puff characteristics (*i.e*., puff volume, inter-puff-interval, *etc*.) when a cigarette is inserted into the device and smoked through a special mouthpiece. Although the CReSS Pocket device has been used in naturalistic settings with smokers [15], it is large and obtrusive, and still relies on the participant to smoke their cigarette through the device. Therefore, CReSS has not been implemented consistently as a way to monitor smoking in the natural environment. A monitoring system that is less obtrusive and allows for more naturalistic smoking would be ideal for detecting smoking events in the smoker’s natural environment. A second system, *mPuff,* measures respiration patterns through chest expansion (detected through a chest strap) to identify smoking events. This system can be worn in the field and directly transmits information to a mobile device [16], though its testing and detection capabilities are still being refined and to date no published record of its performance in relation to direct observation exists. A third system, PACT [17,18], uses sensors at the wrist and chest, and has similar algorithmic and hardware features to mPuff, however currently published reports only reflect its performance with respect to electromagnetic interference in the field; limited information about its performance with respect to concomitant measurement of smoking events is available.

Though remote monitoring systems for the detection of smoking hold great promise for smoking research and treatment, there are several notable concerns in the development and feasibility of remote monitoring technology. One of the most important requirements of a tool used to detect physical activities, such as smoking movements, is that the device itself should not change the nature of the activity [19]. Weight, size, cables attached to the device for data transmission, and where and how the device is placed on the participant’s body can directly affect the participant’s freedom of movement. Although there are many electronic devices to capture kinematic data from participants, many of them are heavy, and require cables and additional equipment to transfer data from the sensors, thus diminishing the goal of devices being unobtrusive.

Joint work between the University of Rhode Island and Rutgers University has recently led to a prototype technology that uses inertial sensors (accelerometers, electrogoniometers and similar hardware) for measuring smoking events. Inertial sensors are small devices that can be used to track movements. They can be applied to limbs of the body and generate raw measurements reflecting tri-axial accelerations, angular velocities, and the relationships to one another. A unique set of algorithms has been developed that combine these signals into a movement detection system [20]. When this technology was initially developed, non-smoking participants were outfitted with small, lightweight body sensors on the arm and wrist, and they mimicked smoking movements. The signals were then sent wirelessly to a handheld computer, such as a smartphone or tablet, where they were processed to detect sham smoking events. Although this methodology overcomes a number of the concerns outlined above, and initial development and validation of the devices and algorithms was promising, actual smoking behavior has never been tested with this system. Therefore, the purpose of the current study was to further validate and formalize the procedure for training the algorithm to differentiate smoking events from non-smoking events with actual smokers in a laboratory setting. The current study also aimed to determine the minimum number and location of kinematic sensors needed to detect smoking events, as well as compare detection accuracy of two different algorithms at identifying smoking events.

## 2. Method

### 2.1. Participants

Smokers (*n* = 6) were recruited from unrelated, on-going or recently completed studies at the Behavioral Pharmacology Research Unit (BPRU) at Johns Hopkins University School of Medicine, where one co-author (EAM) held an appointment. To qualify for the study, participants had to be between the ages of 18–100, report smoking at least 10 cigarettes per day, provide a breath CO sample of >10 parts per million (ppm; piCO+ Smokerlyzer, Bedfont Scientific Ltd., Maidstone, Kent, UK), report smoking for >5 years, and be literate in English. Qualifying participants provided informed consent prior to engaging in study activities. All procedures were approved by the Institutional Review Board at Johns Hopkins University School of Medicine.

### 2.2. Equipment

**Figure 1:**
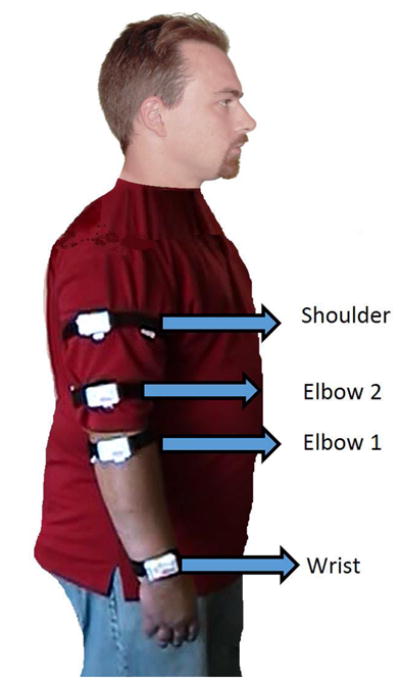
Each white rectangle shows the placement of one Shimmer kinematic sensor on a participant’s arm during the session. Participants 1–3 were outfitted with all four sensors as shown, whereas Participants 4–6 were outfitted with only the Elbow 1 and Wrist sensors on each arm, rather than just the smoking arm. The reference coordinate frame is also shown.

Shimmer^™^ kinematic sensors [21] were used for collecting linear acceleration (with accelerometers) and angular rate (with gyroscopes) values at 50 Hz, capturing arm movements made by the participant during the session. This information was transmitted wirelessly to a destination node connected to an android tablet. The kinematic sensors had a tri-axial accelerometer MMA7260Q made by Freescale and were capable of sensing accelerations ranging from ±1.5 g, ±2 g, and ±6 g, where *g* = 9.81 m/s^2^. Linear acceleration output of the accelerometer is the vector addition of gravity and acceleration of the sensor due to motion. There was also a 3-axis gyroscope board with a full range of ±500 degrees/s. For the first three participants, four sensors were placed on the arm as shown in Figure 1. Based on the results of the first three participants, it was concluded that placing one kinematic sensor on the elbow and one on the wrist was adequate. Consequently, for Participants 4–6, two sensors were placed on each arm (rather than just the smoking arm as before). According to this placement, *+x* axis of the sensor coordinate frame was towards the fingers of the participant, *+y* was aimed at the left side of the arm and *+z* was pointing out from the outside of the arm. A digital video camera, manufactured by Canon (Model FS300) was used in all sessions to record smoking events and to validate the results generated by the kinematic sensors. Based on video recordings, smoking and non-smoking labels were identified by human observers. Training labels for algorithms were randomly chosen over smoking and non-smoking labels by the algorithms.

### 2.3. Procedure

Study procedures were conducted at the Johns Hopkins Bayview Medical Center at the BPRU research facilities. Participants enrolled in the study completed a demographic and psychosocial history questionnaire and the Fagerström Test for Nicotine Dependence (FTND; [22]). The laboratory smoking session lasted for 3.5 hours and required one visit for each study participant. When participants first arrived to the lab, they were outfitted with four kinematic sensors using a velcro strap over their clothing (as shown in Figure 1) and then smoked the first of six cigarettes of their preferred brand. We encouraged participants to sit near a ventilation fan while smoking; however, they were not required to sit in one position while smoking or between cigarettes (*i.e*., they could engage in other activities simultaneously such as reading magazines and talking on the phone). We wanted their smoking environment to resemble their natural environment as much as possible, but in a controlled way at this stage in the research so that we could closely monitor their behavior via video camera. We also required a minimum 20-min inter-cigarette-interval to allow participants time to engage in non-smoking activities during the session for comparison (e.g., reading, talking on the phone, playing cards, eating, walking around). Details of the activities and movements that participants engaged in are described more in Section 3.4.

At the end of the session, participants completed an Acceptability Questionnaire that asked about the tolerability of wearing the kinematic sensors during the session. The session was videotaped so that the data collected from the sensors could be compared to that which was directly observed in the video, which has not previously been done. Six participants completed all study procedures. Two of the six participants smoked cigarettes from the CReSS Pocket topography device (Borgwaldt KC, Inc., Richmond, VA, USA) to obtain specific puffing characteristics, such as puff volume, duration, *etc*. Use of this device substantially modified the way in which participants moved their arm and wrist while smoking each cigarette, as detected by the kinematic sensors, therefore, participants using the CReSS Portable device were excluded from the present analyses. Data are only described for the four participants who smoked naturally during the laboratory session.

### 2.4. Algorithms

The best way to characterize arm movements specific to smoking was to identify the *sequence of puff movement (SPM)* durations. One instance of an SPM was defined as an upward hand movement with the cigarette towards the mouth, placing the cigarette in the mouth and inhaling, which was then followed by a downward hand movement. Since linear acceleration is the vector addition of gravity, plus acceleration due to movement, the acceleration data collected during an SPM characterized both movement (upward or downward) and gravity vector (during inhalation). To detect the occurrence of an SPM, as well as the time in-between puffs (*i.e*., inter-puff-interval) from the raw data collected from the kinematic sensors, two approaches were developed. The first approach employed a supervised learning method based on Support Vector Machines (SVMs). The second approach was based on edge detection in de-noised linear acceleration data.

After the algorithms in both approaches detected a series of SPMs, the whole cigarette smoking events were identified by grouping consecutive SPMs. If the time difference between two consecutive SPM events was large (*i.e*., >10 min), they were separated into different cigarette smoking events. Otherwise, they were grouped into the same smoking event. Finally, cigarette smoking events that had lower SPM counts than the minimum count parameter, which was set to 2, were discarded.

#### 2.4.1. SVM-Based Approach

There has been considerable research on activity recognition using kinematic sensor data with different approaches such as decision trees [23], Bayes classifiers [24] and neural networks [25]. These studies have focused on ambulation posture and activities, such as bicycling. Notably, there is a difference between an activity and a movement that comprises an activity. Smoking a cigarette is an activity whereas taking an individual puff from a cigarette is a movement. Our previous work [20] focused on movement classification using the SVM-based approach. Shortly after the experiment detailed by Varkey and colleagues, another study using devices to track smoking was conducted, however, it relied on a different approach [18].

SVM is one of the most widely used algorithms, particularly for the purposes of recognizing patterns in the data, as well as for classifying text and conducting regression analyses. The basic SVM approach takes a set of data and predicts, for each given input, which of two possible classes form the output, making it a non-probabilistic binary linear classifier.

**Figure 2:**
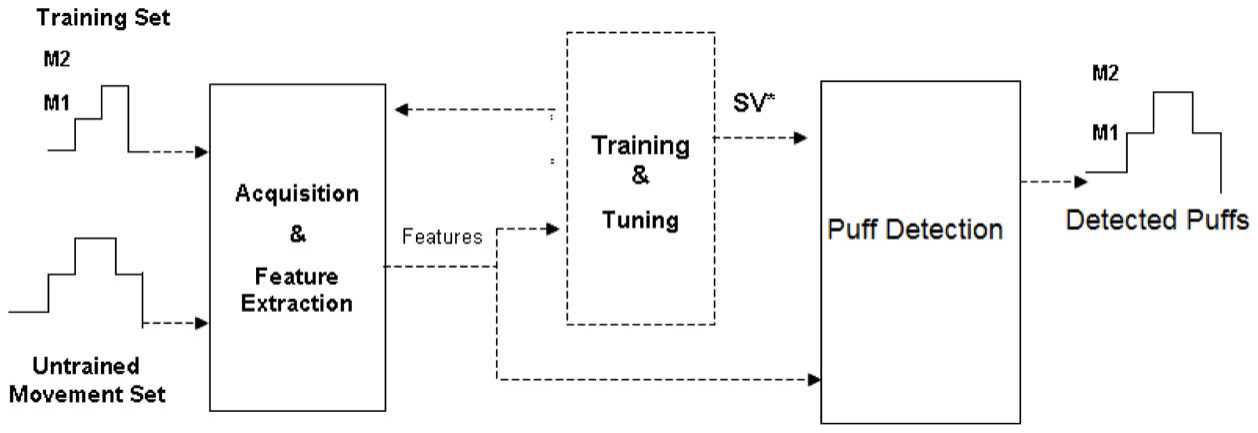
Training and Puff detection for the Support Vector Machines (SVM)-based approach.

Given a set of training examples (e.g., combinations of statistical features extracted from raw kinematic sensor data), each marked as belonging to one of two categories (for the present case the categories were smoking *versus* not smoking), the SVM training algorithm assigns the novel input into one or the other category. Because this algorithm was trained with examples prior to being ready for classification, this approach is a type of supervised learning algorithm. The training examples comprised the features (e.g., mean value, maximum values, *etc*.) that were calculated over a portion (window) of kinematic data. By moving this window over the kinematic data and running the classifier, the algorithm tried to detect SPMs. A block diagram illustrating this training and detection with the SVM approach is shown in Figure 2.

The specific elements of our SVM approach were as follows. Let **q(*t*)** denote the raw accelerometer and gyroscope values at time *t* and **Q(***t*) is a p-dimensional vector that is composed of the features extracted from the **q(*t*)** in time interval between *t* and *t* + *w*. The *w* represents the window size, which is a parameter of our algorithm. Therefore, every time interval during a session can be represented as a point p dimensional space and each of those points belong to one of the classes (smoking or not smoking). In SVMs, the objective is to find a (p-1) dimensional hyperplane that separates the classes. A hyperplane can be defined as,

(1) F(x)=ax+b

where ***x*** is the vector to be recognized, **a** is the normal vector to the hyperplane and **b** is the offset from the origin of the space. Therefore, the purpose of the training in SVM is to find **a** and **b**.

Based on past experience in activity and movement classification with SVM [18,20], radial basis function kernel was used to tune the SVM parameters and the cumulative misclassification ratio was used as an index; this is the ratio of total misclassification of movements to total activity time. We calculated mean, standard deviation, maximum, minimum, peak to peak, root mean square and correlations between axes of the data within the window to generate features.

The window-based algorithm that was used was composed of two windows, a main window and a small window. Although the main window and small window had the same *window size* (*w*), the small classification window was shifted by *w/2* within the main window two times. Four different window sizes (5, 10, 15 and 20 s) were evaluated and the best results were observed with the 10-s window size, based on the Receiver Operator Characteristic (ROC) analyses reported in Section 3.3. For each shift of the small classification window the confidence was calculated. Therefore, the recognized movement fell within the classification of the window resulting in the best confidence.

#### 2.4.2. Edge-Detection-Based Approach

With this approach, the raw data were first preprocessed to eliminate noise and gravity components. To filter noise, an equiripple FIR lowpass filter was used at 1 Hz. This filter was not used in the SVM approach because SVM does not draw on raw data but rather uses statistical features (e.g., mean, min and max) from raw data that are less sensitive to noise. We assumed that gravity components between consequent samples were the same. Although, the previous acceleration sample has both gravity and the acceleration-due-to-motion components, subtraction of the previous sample from the current sample is an approximation to gravity elimination. Next, a basic edge-detection algorithm was applied. A kinematic sensor that is in motion will produce increasing or decreasing acceleration values over time. A fixed sensor, on the other hand, will produce a constant acceleration (due to gravity) plus random noise (due to measurement errors). The basic edge detection algorithm defined significant increases and decreases as edges. Every rising or falling edge of the filtered signal corresponded to a hand movement. Finally, upward and downward hand movements were utilized to model an SPM. To model SPM, minimum and maximum SPM duration parameters were defined by observing the SPM durations from video recordings. When the time difference between the participant’s upward and downward hand movement coupled together was lower than the minimum SPM duration, or higher than the maximum SPM duration, that movement was assumed to be a false (impossible) event and it was not included.

Since edge-detection-based methods detect significant increases/decreases in kinematic sensor data, the classification output of the method, Y(*t*), was 1 (representing an upward or downward arm movement) if the difference of consequent kinematic sensor data samples, t, were greater than a certain threshold, *th*. Otherwise, the edge-detection-method output was 0 (shows that the algorithm did not detect an upward/downward movement). Therefore,

(2) $$Y(t)=\left\{\begin{array}{c} 1,q(t)-q(t-1)>\mathrm{th}\\ 0,\text{otherwise} \end{array}\right\}$$

Since the signal was already filtered, only a difference that was greater than *th* was detected between concurrent samples due to movements. Although edge-detection method could generate too many 1 s for small values of *th* (detecting very small movements), the algorithm’s performance depended on a correct choice for the value of *th*, based on minimum and maximum SPM duration. As with the SVM-based approach, selection of these parameters were based on Receiver Operator Characteristic (ROC) analyses (described in Section 3.3). The accuracy of the algorithm came from its ability to filter erratic movements (e.g., playing with hair). In order for an erratic movement to be counted as an SPM, it needed to be similar not only in amplitude, direction, and duration but it also needed to occur for longer than the minimum SPM duration.

### 2.5. Detecting Puff and SPM Durations

To identify puff and SPM durations using the kinematic sensors, first we examined different sensor combinations to determine the best locations on the arm, as well as the minimum number of sensors that would be needed to detect such movements. We also investigated different values for two training parameters, with 10 different feature extraction methods for the SVM-based-approach, and 21 different thresholds for the edge-detection-based approach. Next we chose the best configuration, which was comprised of the feature set, sensor combination, and training parameter or threshold value, and the minimum and maximum SPM duration that produced the best performance, to use in further analyses (e.g., to determine puff and cigarette levels).

Determining the best combination involved using a Receiver Operator Characteristic (ROC) analysis, with direct observation serving as the *Ground Truth* for determining when each cigarette was smoked during the session (verified by the video recordings). The ROC curve is a graphical plot illustrating the performance of a binary classifier system as its discrimination threshold varies. It was created by plotting the fraction of true positives out of all positives from the ground truth (TPR = true positive rate) *vs*. the fraction of false positives out of all negatives from the ground truth (FPR = false positive rate), at various threshold settings.

Next, the focus was on SPM level analysis where the methods were evaluated by comparing the results with the Ground Truth in terms of the number of puffs taken, detected SPMs, and duration of SPMs for each participant. Duration between SPMs and inter-puff durations were also examined. A second analysis was then deployed at the level of the cigarette. By combining consecutive SPMs, we identified when a cigarette was smoked. Similar to the SPM level analysis, the performance of methods were evaluated by comparing the number of cigarettes actually smoked (all participants smoked a total of 6 cigarettes), as well as the duration of time taken to smoke each cigarette, based on Ground Truth compared to that detected by the algorithms. All ground truths were based on observing the video recordings from each participant’s session.

To assess agreement between the ground truth for number of puffs for each cigarette and the duration of time it took to smoke each cigarette, independent observer ratings were compared based on review of participant session videos. Inter-observer agreement (IOA) for puffs and duration of each cigarette was calculated by dividing the smaller number by the larger number and multiplying by 100 to obtain a percentage agreement. Agreement percentages were then averaged for each cigarette smoked.

## 3. Results

### 3.1. Participants

Individual participant demographics, past, and current smoking characteristics are shown in Table 1. On average, study participants (*n* = 6) were 40.3 (SD = 10.2) years old. The majority of participants were male (83%) and White (83%). Participants smoked an average of 19.6 (SD = 6.0) cigarettes per day, and had been smoking regularly for an average of 20.3 (SD = 7.5) years. Nicotine dependence scores, determined via the FTND, averaged 5.2 (SD = 1.9), and prior to their laboratory session, participant breath CO samples averaged 13.7 ppm (SD = 5.3). Generally, participants were moderate to heavy smokers with a substantial history of smoking.

### 3.2. Smoking Characteristics

Two participants smoked cigarettes from the CReSS Pocket smoking topography device, and were excluded from the the following analyses because the CReSS device changed wrist and arm movements detected by the kinematic sensors. Smoking characterstics, as directly observed and calculated via the video-recordings, are shown in Table 2. Across six cigarettes, participants averaged 9.2 (SD = 3.9; Range = 4–21) puffs per cigarette. This average does not include the initial puff that was used to facilitate lighting the cigarette. Participants paused for approximately 42-s between individual puffs (SD = 21.3; Range = 13–110-s) and spent approximately 367-s (SD = 79; Range = 224–506-s) smoking each cigarette during the laboratory session. Agreement ratings from two independent observers were 92.8% and 94.3% for puffs per cigarette and duration to smoke a cigarette, respectively. For the remainder of the smoking measures, each participant is described separately.

### 3.3. ROC Analyses

**Figure 3:**
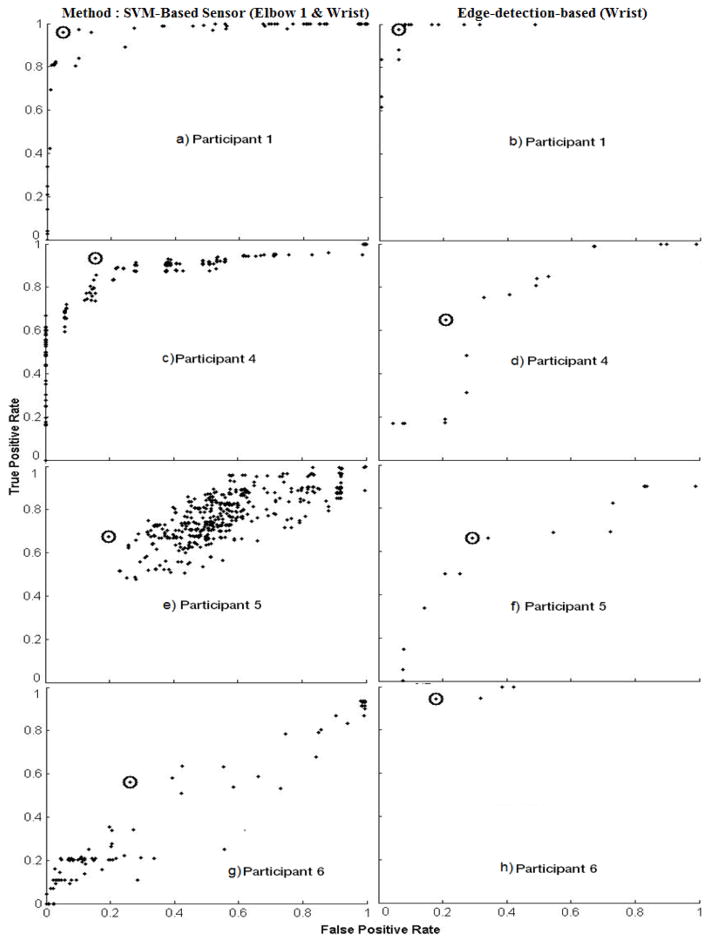
Receiver Operator Characteristic (ROC) Curves for each participant with SVM-based (left column) and Edge-detection based methods (right column). Every data point represents a different configuration feature set. The circled points indicate the configurations that produced the best results.

Figure 3a, b show the ROC curves for the SVM-based approach and edge-detection-based methods for Participant 1, respectively. The best configurations used in cigarette and SPM level analyses are indicated by the circled point on the figure. The best configuration had a high TPR (>0.95) and a low FPR (<0.07) for both of the algorithms. This high TPR and low FPR leads to success in SPM level analysis. Participant 4 (Figure 3c, d) also had the best configuration for the SVM-based approach and showed a high TPR (>0.9) and an intermediate FPR (<0.2). On the other hand, the best configuration for the edge-based algorithm resulted in lower performance for TPR (>0.6) and FPR (~0.2). It should be noted, however, that the SPM durations detected by the edge-detection-based approach were similar to Ground Truth identified in the video. Figure 3e, f show the ROC curves for Participant 5. The best configuration for both SVM-based and edge-detection-based approaches resulted in intermediate TPR (~0.7) and FPR (~0.2). Finally, Participant 6 (Figure 3g, h) showed the best configurations for both methods and had FPR around 0.2, the edge-detection-based algorithm produced a higher TPR (>0.95) then the SVM-based approach (>0.6).

### 3.4. Puff Duration and SPMs

**Figure 4:**
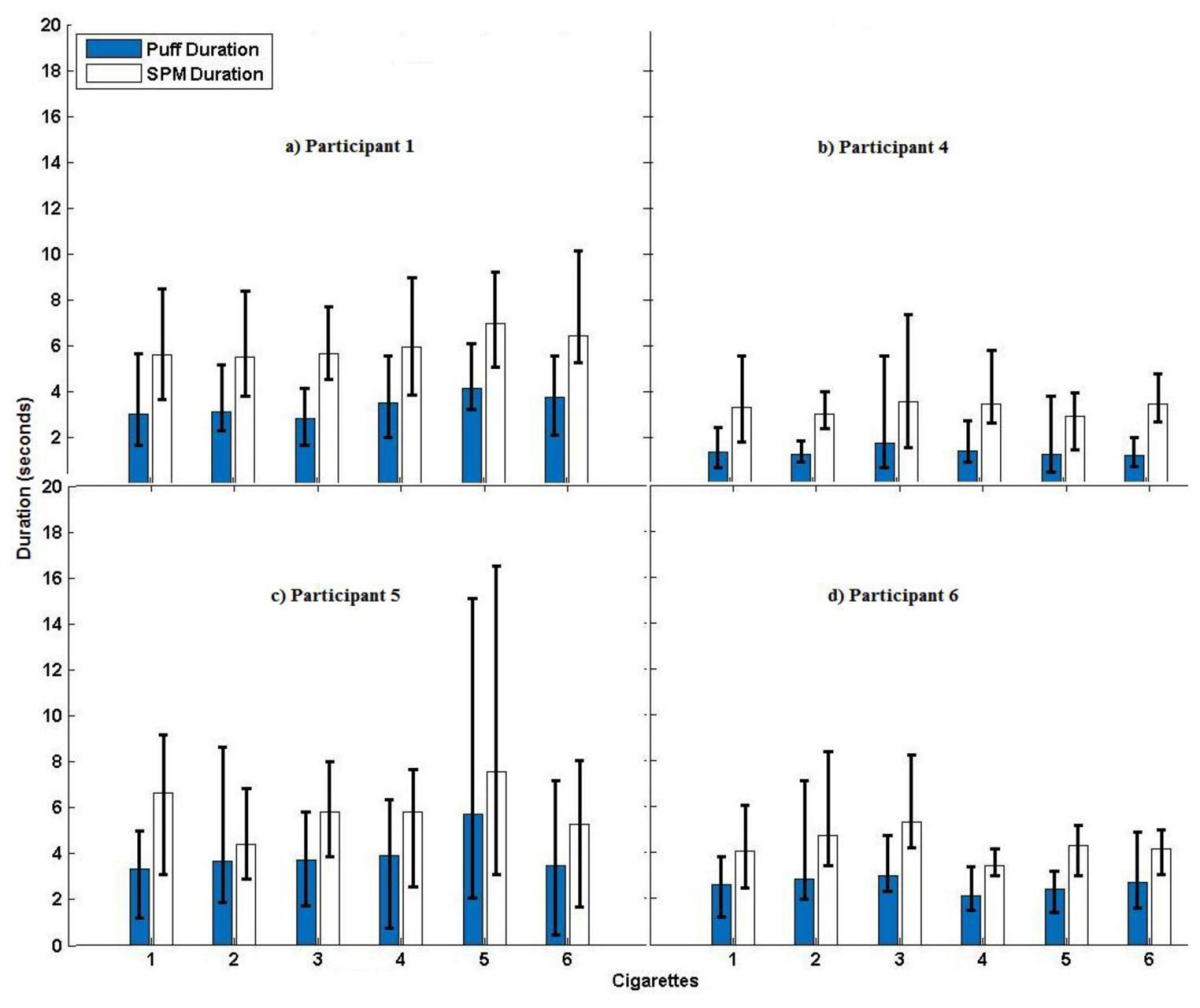
Mean Sequence of Puff movement durations (SPM; blue bars) and mean Puff durations (white bars) are shown for each participant across each of the six cigarettes smoked during the session (determined by watching video recordings of participants smoking during the session). Error bars represent minimum and maximum values.

Figure 4 shows the Puff and SPM durations of each participant based on the video recordings. SPM durations are expected to be longer than puff durations as they capture the entire arm movement of the puff (raising the hand to the mouth, inhalation, and then lowering the hand). Participant 1 (Figure 4a) had SPMs averaging 6-s (Range = 4–8-s), and displayed longer and less variable puff durations, relative to the other participants. Participant 4 (Figure 4b) displayed short SPM durations averaging 3-s (Range = 2–8-s), with intermediate variations across SPM durations. Additionally, during the video Participant 4 was seen talking and reading magazines. Although he did not engage in many erratic or unidentifiable actions, he did use his smoking hand to support his head at times. Participant 5 (Figure 4c) had average SPM durations of 6-s (Range = 2–16-s), showing greater range than the other participants. Additionally, during the video the participant was seen engaging in other activities (e.g., reading, taking notes) and performed more erratic and difficult to classify movements, such as scratching her head and playing with her hair. Finally, Participant 6 (Figure 4d) had an average SPM duration of 4-s (Range 3–8-s). His SPM characteristics were similar to Participant 4 but based on the video observation, he also performed a number of erratic and difficult to classify actions (e.g., scratching head).

**Figure 5:**
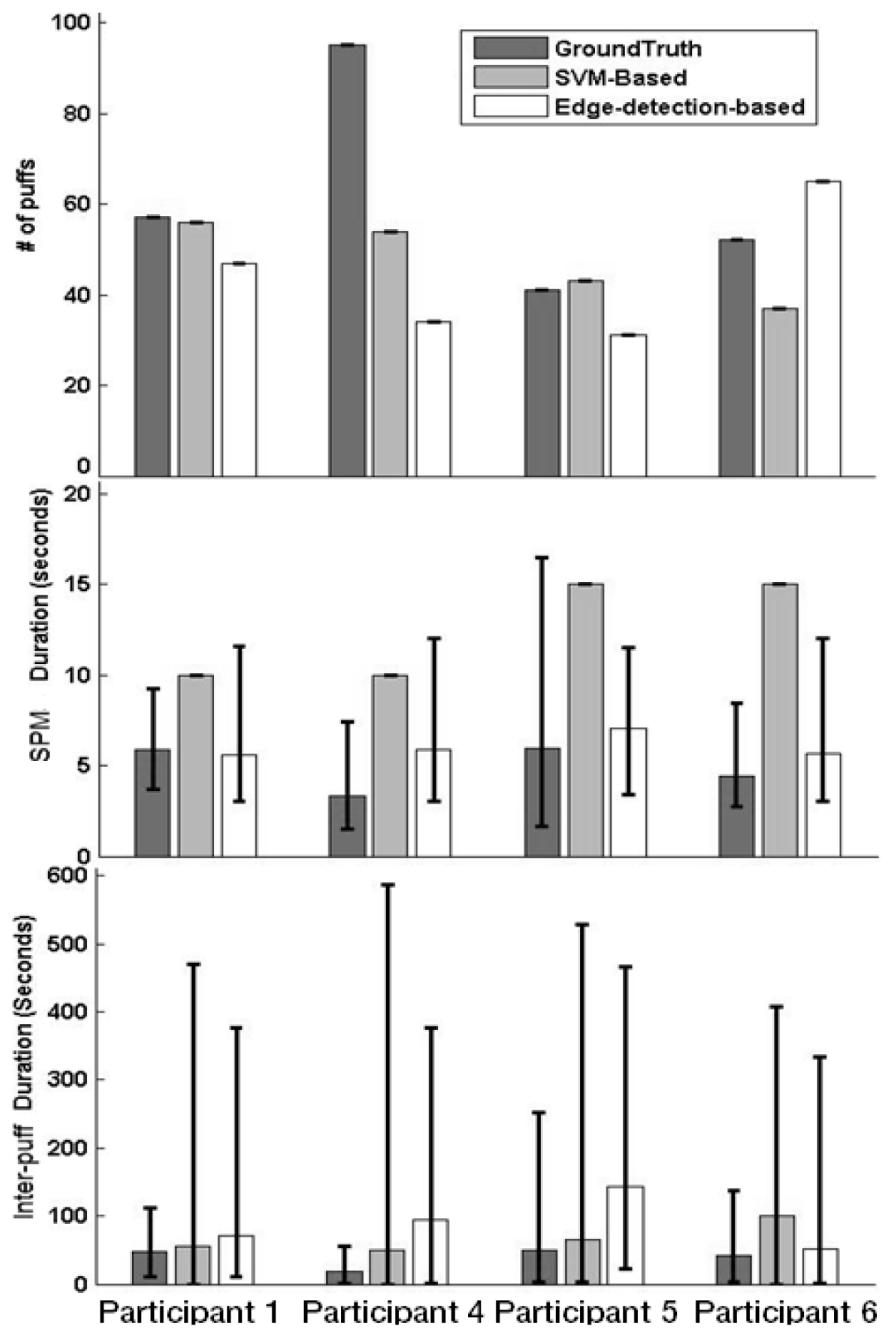
Number of SPM events (top row), SPM durations (middle row) and Inter-SPM durations (bottom row) from Ground Truth (based on video; dark grey bars), SVM-based (light grey bars) and edge-detection-based methods (white bars) for each participant. Error bars represent minimum and maximum values.

For Participant 1, Figure 5 shows that the number of puffs and SPMs reported by the algorithms closely matched. Table 3 indicates that the edge-detection-based approach correctly detected 45 of 57 puffs (*i.e*., true positives) and only resulted in two puffs that did not actually occur (*i.e*., false positives). Additionally, Figure 5 shows SPM durations from the edge-based algorithm were close to Ground Truth. It should be noted that the SVM-based method could only produce a fixed SPM duration, because granularity was restricted by the duration of the 10-s window chosen, which was a training parameter. Due to this restriction, the SVM-based algorithm could only generate 10-s SPM durations. Figure 5 shows that the number of SPMs, SPM duration, and inter-SPM intervals were well detected for Participant 1. For Participant 4, Figure 5 shows that as the duration of SPMs became shorter, the number of missed SPMs increased. Figure 5 also shows that for Participant 4 the number of puffs detected by both algorithms were less than the values of the Ground Truth. Table 3 shows that the SVM-based and edge-detection-based approaches captured 22 and 20 of the 95 puffs correctly, whereas the algorithms identified 53 and 16 puffs that did not occur (*i.e*., false positives), respectively. For Participant 5, although the SPM level analyses in Figure 5 show that both the SVM-based and the edge-detection-based approaches resulted in a similar number of SPM’s against the Ground Truth, a high number of false positives is shown in Table 3 which explains the reason for the intermediate TPR and FPR. Finally for Participant 6, again SPM level analyses show that both approaches resulted in a similar number of SPM’s against the Ground Truth, Table 3 shows a higher number of false positives than true positives.

### 3.5. Cigarette-Level Analyses

Because cigarette events were contracted from consecutive SPM events that were detected by the algorithms, cigarette level analyses depended on SPM level analyses. In other words, if the algorithms produced low performance on the SPM level analysis (e.g., did not detect an SPM), then it was not possible to construct the occurrence of a cigarette either.

**Figure 7:**
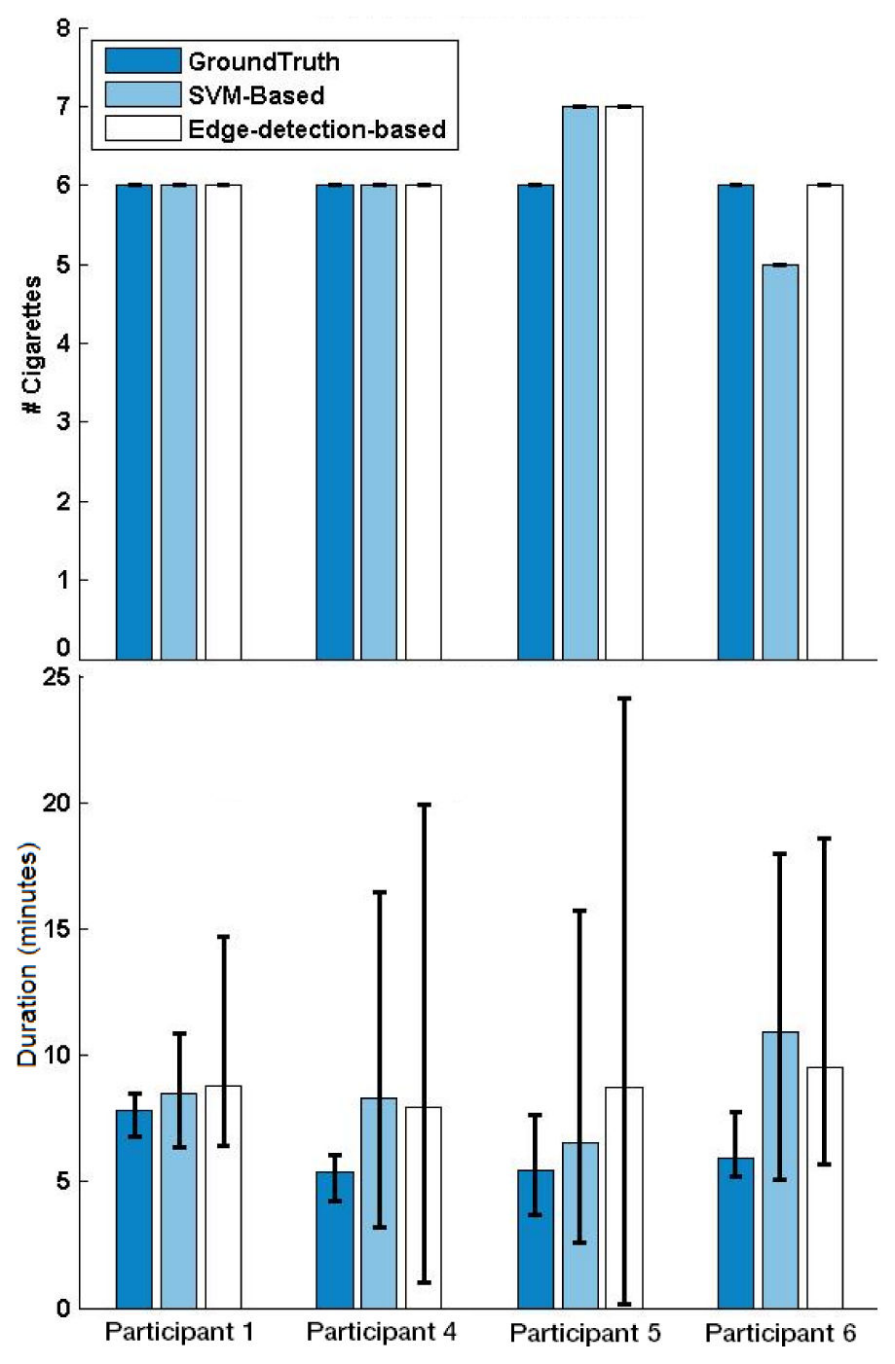
Number of cigarettes (top row) and cigarette durations (bottom row) from Ground Truth (based on video; dark blue bars), SVM-based (light blue bars) and edge-detection-based method (white bars) are shown for each participant. Error bars represent minimum and maximum values.

Figure 7 shows results from the SVM-based and edge-based-detection approaches for number of cigarettes detected, as well as the duration taken to smoke a cigarette. Both the SVM-based and edge-detection-based approaches correctly identified all six cigarette smoking events for Participant 1. Table 4 also indicates that for Participant 1 all cigarettes that were observed in the video were also identified by the algorithms (*i.e*., true positives). Based on Figure 7, there was a small time difference (less than 1 min) in the average duration detected per cigarette. When smoking events were investigated at the cigarette level for Participant 4, the number of cigarettes was correctly detected by both algorithms (Figure 7 and Table 4). For Participant 5, there were a high number of false positives at the SPM level, which led to an overestimation of the number of cigarettes smoked, regardless of the algorithm used. Finally, for Participant 6, Figure 7 shows that the edge-detection-based approach identified six cigarette smoking events, all of which were true positives (also see Table 4).

**Figure 8:**
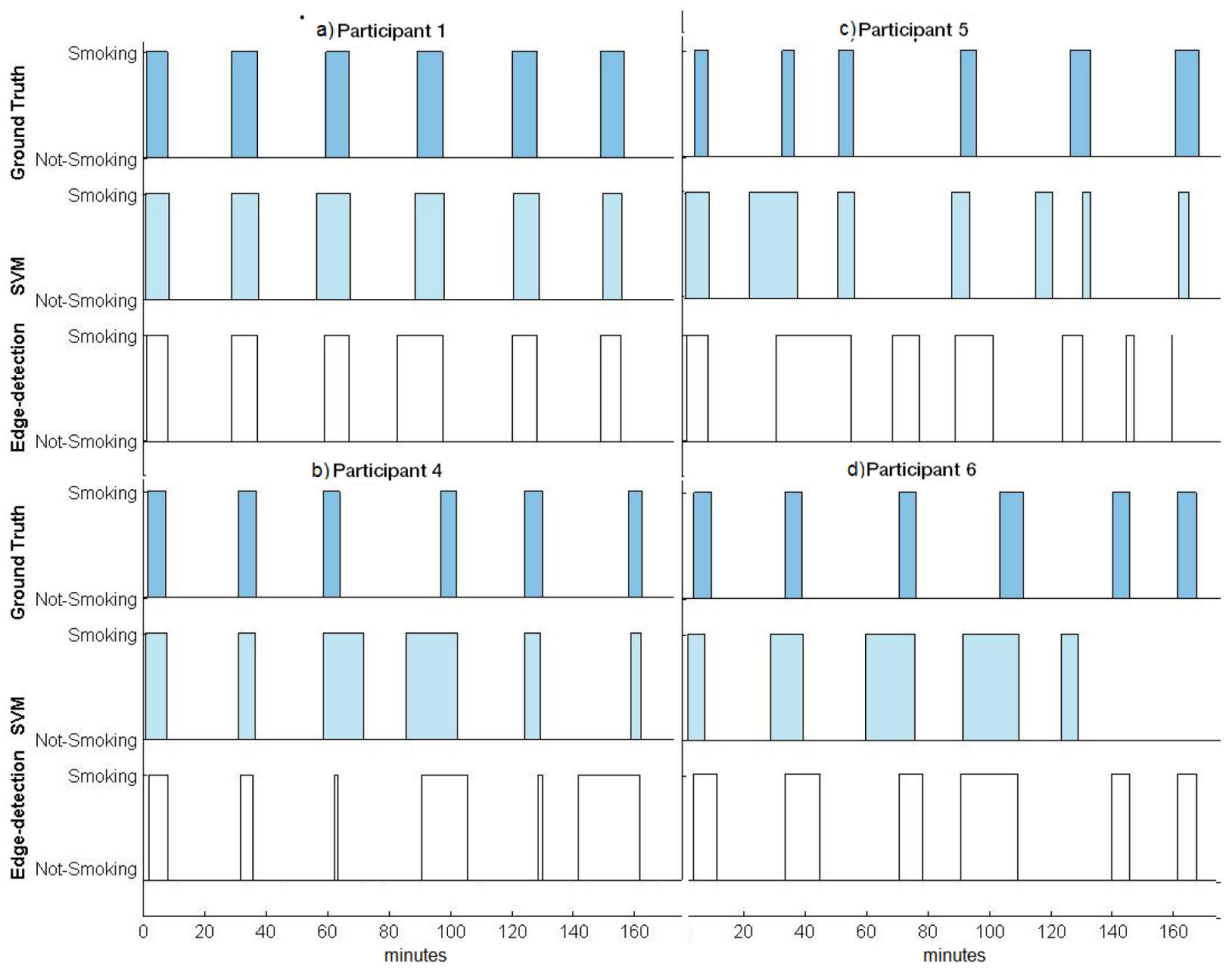
Timing of cigarettes from Ground Truth (based on videos; dark blue bars), SVM-based (light blue bars) and edge-based methods (white bars) are shown for each participant. Each rectangle represents a cigarette-smoking event in time.

Figure 8 shows the time during the session when each cigarette was smoked based on the Ground Truth and algorithm detection. Based on review of the detailed cigarette timings shown for Participant 1 (Figure 8a), and comparing this to what was seen in the video, the slight difference observed occurred due to non-smoking arm movements that were consistently present with each cigarette smoked (*i.e*., picking up a lighter when initiating each cigarette smoking event). For Participant 4 (Figure 8b), the differences between the cigarette durations were primarily a result of differences across measures during the third and fourth cigarette with the SVM-based method, and the differences across measures for the fourth and sixth cigarettes with the edge-detection-based method. Observations made in the video showed that the errors were again due to arm movements that resembled taking a puff from a cigarette, such as head scratching before and after the cigarette was smoked. For Participant 5 (Figure 8c), the edge-detection-based approach became less accurate as the participant started to engage in other, non-smoking and difficult to classify activities. The SVM-based approach performed better under these circumstances than the edge-detection-based approach. Finally, for Participant 6 (Figure 8d), cigarette durations detected by the edge-detection-based method were more consistent with those observed in the video than the durations detected by the SVM-based approach. The SVM-based approach detected five cigarette smoking events, one of which was a false positive. This performance error with the SVM-based method was due to poor performance at the level of detecting individual SPMs. Cigarette events for Participant 6 were detected from both methods.

### 3.6. Sensor Acceptability

Following laboratory procedures, participants completed a brief acceptability questionnaire on various aspects of the sensors. Using a 100-point visual analog scale, participants (*n* = 6) rated the sensors as comfortable to wear (mean = 77, SD = 29) and acceptable to wear for extended periods outside of the laboratory (mean = 70, SD = 33). Participants also responded that they would be likely to wear the sensors outside of the laboratory (mean = 76, SD = 26). Five out of six participants felt that the sensors may be able to help them quit smoking, if they received feedback during a quit attempt. Finally, all six participants said they would wear the sensors for extended periods outside of the laboratory if they thought it might help them to quit smoking.

## 4. Discussion

Our overall goal in this pilot study was to test the feasibility of a novel smoking detection system based on unique smoking-related arm movements. This study was a critical step in the development of a sensor-based system for detecting smoking in more natural settings. Although this study was conducted in a controlled laboratory environment, participants were free to move around and engage in other behavior at the same time that they were smoking (e.g., reading magazines, talking on the phone, playing cards, *etc*.), which brings this program of research closer to testing in the less-controlled natural environment. Although, participants were asked to sit near a vent, we believe algorithms will work when the participants are walking and smoking if the training set is properly chosen. This study was also an important step in the process by helping identify how different hand gestures, head, and upper body movements, affected the performance of the algorithms. Finally, this study helped determine the ideal sensor configuration and algorithmic approaches for further testing in more naturalistic and outpatient settings, a logical next step in this program of research.

The system performed remarkably well with respect to identifying when a whole cigarette was smoked. Results for identifying individual puffs were more variable and depended on characteristic movement patterns across individual subjects, as well as the algorithmic approach that was being used. We are confident that in the future, with further testing and refinement, this novel sensor-based movement detection system for identifying smoking events will yield useful information for researchers and clinicians. Smoking research, as well smoking cessation treatment development and implementation, could benefit from systems that monitor smoking events in the natural environment. Studies utilizing remote monitoring methods would alleviate a great deal of burden placed on participants and research staff, while also potentially improving the accuracy of the data and outcomes because objective measures of individual smoking events could be detected, as opposed to self-reports or more global biochemical measures that are currently relied upon today. Of course, there is still a great need to increase the fidelity and efficacy of sensor-based systems [26]. The current study is a step in this direction by rigorously testing a monitoring system in the laboratory.

Specific consideration of each algorithm’s performance is important to guide future work. Based on the results of each individual participant, both of the algorithm detection methods (Edge-based and SVM-based) performed well (66%–100% and 83%–100%, respectively) with regard to the cigarette level analysis. The performance of the edge-detection-based algorithm at the cigarette level may be more promising. With the edge-detection-based algorithm, there were false positives only for Participant 5. Additionally, the SVM approach was also less accurate in detecting individual puffs. The SVM approach detected 17%–29% of puffs, and the false positive count was as low as 32 (median false positive = 44). On the other hand, the Edge-detection-based approach was more effective at detecting puffs than the SVM-based method, detecting 7%–79% of puffs, with a false positive count as low as 2 (median false positive = 22).

Performance at the cigarette level was very good with all but Participant 5. This participant engaged in a number of non-smoking activities, such as reading or speaking, during the smoking period which caused a greater degree of variability in the inter-puff durations (see Figure 5). This greater variability caused the algorithms to group puffs into separate shorter duration puff groups. The shorter puff groups affected not only the overall cigarette duration, but also the cigarette count. The shorter puff groups also led the algorithms to detect a greater number of cigarettes than really occurred at times, *i.e*., resulting in false positives. Another possibility is that the shorter puff groups may not have contained enough puffs to comprise a full cigarette, thereby decreasing the number of cigarettes detected, resulting in a higher occurrence of false negatives. Overcoming these problems entails training the algorithms to detect smoking events at the level of the cigarette, as opposed to the level of the puff by developing cigarette construction parameters. Namely, it will be important to train the algorithms to identify the time difference between consecutive SPMs and identify the minimum number of SPMs needed to construct a cigarette. Future studies should focus on continuing to refine the two algorithmic approaches to best capture SPM and puff durations such that they can accurately distinguish between cigarette smoking arm movements from non-smoking arm movements, especially in situations where the smoker engages in a number of erratic arm-movements that resemble SPMs. Below is a more detailed account of our future plans for our sensor configuration and the algorithms, followed by a short summary of potential applications of this approach to smoking research and smoking cessation intervention.

### 4.1. Suitability of Sensor Configuration

Participants 1–3 were outfitted with four kinematic sensors. When conducting the ROC analyses to determine the best configurations, it was discovered that only the elbow2 and wrist sensors were needed to adequately to characterize the movement, because the elbow 1 sensor produced similar data with the wrist sensor, and the shoulder sensor produced similar data with the elbow2 sensor. Based on these results, only two sensors were used for the remaining participants (4–6), one on the elbow and one on the wrist. The SVM-based-approach performed best when the data from both sensors were used, whereas the edge-detection-based-approach only required the wrist sensor. This finding is important as fewer sensors may increase the comfort and acceptance of wearing these sensors outside of the laboratory. Acceptability questionnaires showed that participants found the sensors to be comfortable and were enthusiatic about wearing them outside of the laboratory if there was the potential for them to help with a quit attempt. It should be noted that the current devices, hardware, velcro straps, *etc*. are a prototype of this sensor technology, and will become smaller, lighter, and even less invasive through future iterations.

### 4.2. Future Algorithmic Challenges

Central to our project became the experimental contrasts of two algorithms, SVM-based and Edge-detection-based. Given this, we discuss key considerations in light of this comparison in some detail.

The video observations revealed that there was individual variability across participants with regard to non-smoking arm movements, which led to differences in the performance of the algorithms across participants and measures, primarily when it came to detecting SPM and puff durations. For example, the slow and distinct movements of Participant 1 enabled both the SVM-based and the edge-detection-based approaches to achieve excellent detection abilities that outperformed the detection abilities of the two approaches when applied to the other participants. Also, as puff durations became shorter, it became more difficult to distinguish puff events from other arm movements, such as head scratching. For example, Participant 5 exhibited shorter puffs, as well as more erratic non-smoking arm movements, and this resulted in poorer performance for puff detection even for the SVM-based approach, which trains itself to capture participant specific smoking patterns. Because these erratic, non-smoking arm movements were not repetitive, they were incorrrectly counted as puffs. Therefore, these false positive puff detections presented a problem when attempting to identify smoking events at the SPM level, but not at the cigarette level, regardless of the algorithm used.

Finally, as mentioned earlier, cigarette level performance depended in part on SPM level results. It can be difficult to distinguish puff arm movements from non-puff arm movements (e.g., those that involve touching the head, such as scratching) while the participant is smoking. Nonetheless, although some of the false positive SPMs are currently unavoidable, the whole cigarette recognition remains quite high. Improvements in SPM level would contribute to an improved cigarette level performance. This would be an important advancement, as cigarette count tends to be the parameter of greatest interest. Improvements at the puff recognition level are important for detection, but these improvements would need to be motivated largely by scientific interests in more specific puffing characteristics, such as puff volume and duration, which have been shown to quantify toxin exposure [27]. Also, this level of detection would allow for real-time interventions that could target puffing instances rather than entire cigarette recognition, because sometimes just taking a few puffs from a cigarette (i.e., a lapse) can result in a full blown relapse, and detecting such lapses early would allow interventions to be delivered immediately when they are needed. In light of these considerations, we believe that the following future work is critical to continued improvement of our approach. We are also working on inferential techniques [13] and research designs [19] for the study of smoking movement in clinical trials, as our technology-based work progresses.

#### 4.2.1. SVM-Based Approach

The input to the SVM classifier (*i.e*., the feature set extracted from the raw data) played a critical role in performance of this approach. Future work will be aimed at finding additional features that best characterize the movements (e.g., those that capture the time correlation between different sensor measurements in raw data from one sensor and across multiple sensors). In the current study the accuracy of the SPM duration reported by the SVM-based approach was dependent on the size of the aforementioned window used for classification (e.g., 10-s). Future work should further refine the model to extract the exact SPM durations and, therefore, the puff durations.

#### 4.2.2. Edge-Detection-Based Approach

After the basic edge-detection algorithm recognized upward and downward hand movements, a candidate set of SPMs was identified. However, this set had to be cleaned automatically by the algorithm to eliminate false alarms (e.g., if there was too much/too little time that elapsed between the upward and downward movement) and to obtain actual SPMs. In the current study, parameters to automatically remove false positives (*i.e*., minimum and maximum SPM duration and minimum SPM count) from the candidate set were based on logic alone. Further elimination of these false positives would improve the robustness of the system. Future plans involve developing a machine-learning-based model, which incorporates knowledge of average, maximum, and minimum SPM as well as inter-puff durations (specific to each participant) to eliminate outliers.

Apart from the improvements that can be implemented in the aforementioned approaches, adding another decision method on top of these approaches might also improve the results significantly. These types of decision methods are called “ensemble learning” methods in machine learning [28]. In ensemble learning, multiple hypotheses from different methods “vote” in some fashion to obtain better predictive performance than could be obtained from any of the constituent methods. Using the current analysis as an example, we could run both SVM-based and edge-detection based methods, then get their prediction for a kinematic sensor output for a specific time interval as well as their confidence in the result. Finally, an ensemble learning method choses the best output in real time. The critical part of this approach is determining confidence for SVM-based and edge-detection-based methods.

### 4.3. Summary

The current study provides a systematic replication of our initial proof-of-concept study [20] for sensor-based tracking of smoking movements of the arm and wrist and, more importantly, provides the first pilot results of the system’s efficacy with actual smokers whose movements were unrestricted and who could engage in other behavior (e.g., reading a magazine, talking on the phone, playing with hair, *etc*.) while they were smoking. These data show that two families of machine learning algorithms can be used with a reasonable set of sensor hardware placements to detect cigarette events with high confidence in actual smokers. The current approach was not as effective at the level of detecting individual puffs because of the considerable variability that was introduced by participant-specific non-smoking movements. Nevertheless, we are optimistic that future work will enable us to track, in real-world environments, detailed measurements of smoking topography that has not previously been possible. The kinematic sensor detection system examined in the current study has great potential for improving smoking research and smoking cessation interventions. Future research will further refine the algorithms and aim to improve this detection system, especially as the events occur in the smoker’s natural environment. This system will ultimately measure smoking, while also maintaining the privacy of participants, which is of paramount importance in any remote monitoring system, mHealth technology, or treatment intervention.

## Figures and Tables

**Figure 6:**
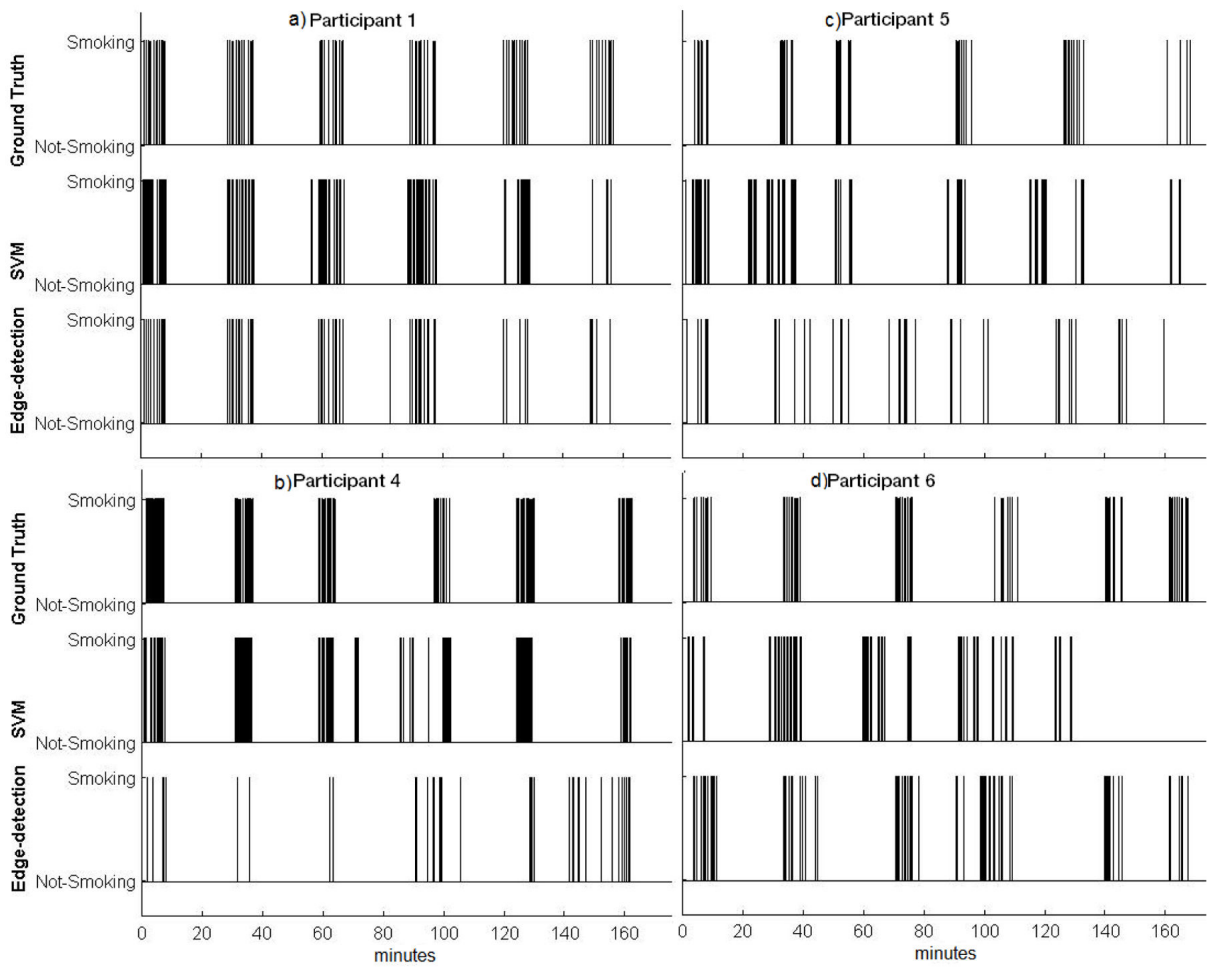
Timings of each SPM detected from Ground Truth (top row for each participant) SVM-based detection (middle row for each participant), and edge-based detection (bottom row for each participant). Each line represents an SPM event in time across each of the six cigarettes smoked during the session.

**Table 1:** Participant demographics and smoking characteristics.

Participant	Gender	Age	Race	Education	Weekly Income ($)	Cigs/day	Years smoking	FTND	Intake CO	CReSS used?
1	M	40	White	Grade school	100–200	30	28	8	24	N
2	M	28	White	Some HS	201–300	12.5	10	4	10	Y
3	M	28	White	HS grad	>100	20	16	3	10	Y
4	M	48	AA	Some College	301–400	20	20	7	12	N
5	F	50	White	Some College	>100	15	30	5	12	N
6	M	48	White	Some HS	201–300	20	18	4	14	N

Race: African American (AA). Education: High School (HS). Smoking Characteristics: Fagerström Test for Nicotine Dependence (FTND; range 0–10), Cigarettes per day (Cigs/day), Carbon Monoxide (CO), Clinical Research Support Systems (CReSS) pocket smoking topography device.

**Table 2:** Average puff number, mean inter-puff interval (s), and total cigarette time (s) averaged across six cigarettes for each participant.

Participant	Puffs	Mean Inter-puff Interval (s)	Total Cigarette Time (s)
1	8.5	51.1	464.0
4	14.8	18.8	323.7
5	5.8	56.0	325.9
6	7.7	44.8	355.0
Average	9.2	42.7	367.2

**Table 3:** Number of true positives and false positives for SPM events for both methods across participants.

Participant	Total # of Puffs (Ground Truth)	True Positives		False Positives	

		SVM-Based	Edge-detection based	SVM-Based	Edge-detection based
1	57	15	45	53	2
4	95	22	20	53	16
5	41	12	3	32	28
6	52	9	30	38	35

**Table 4:** Number of true positives, false positives and false negatives at the cigarette level for both methods across participants. Number of false negatives equals to total number of cigarettes (6) subtracted by true positives.

Participant	True Positives		False Positives	

	SVM-based	Edge-detection based	SVM-based	Edge-detection based
1	6	6	0	0
4	6	6	0	0
5	6	5	1	2
6	4	6	1	0
